# Endurance training increases tmTNF-α and IL-6 levels in several vital organs in rats: relationship with hypoxia markers

**DOI:** 10.3389/fmolb.2026.1815657

**Published:** 2026-05-14

**Authors:** Lukasz Galganski, Jerzy A. Zoladz, Wieslawa Jarmuszkiewicz

**Affiliations:** 1 Mitochondrial Biochemistry Research Group, Faculty of Biology, Adam Mickiewicz University in Poznan, Poznań, Poland; 2 Chair of Exercise Physiology and Muscle Bioenergetics, Faculty of Health Sciences, Jagiellonian University Medical College, Krakow, Poland

**Keywords:** cytokines, endurance training, hypoxia markers, inflammation, organ-specific adaptations

## Abstract

**Background:**

Although endurance training is known to induce systemic adaptations involving inflammatory and hypoxia-related signaling, tissue-specific protein responses remain incompletely understood. Therefore, this study examined the effects of endurance training on inflammatory cytokines and hypoxia markers across multiple organs.

**Methods:**

Male Wistar rats performed treadmill running (30–60 min/day, 5 days/week) for 8 weeks. Protein expression of interleukin 6 (IL-6), transmembrane tumor necrosis factor–alpha (tmTNF-α), soluble TNF-α (sTNF-α), hypoxia-inducible factor 1 subunit alpha (HIF-1α), and lysine demethylase 6A (KDM6A) was assessed in the lungs, liver, skeletal muscle, heart, and brain.

**Results:**

Endurance training increased IL-6 protein levels in the lungs, liver, skeletal muscle, and heart, but decreased them in the brain. It also increased tmTNF-α protein levels in the lungs, liver, skeletal muscle, and heart, but decreased them in the brain; it did not affect sTNF-α protein levels in any organ. Hypoxia markers responded to endurance training in an organ-specific manner: HIF-1α and KDM6A protein levels increased in the lungs, liver, and heart, whereas KDM6A protein levels increased while HIF-1α protein levels were unchanged or decreased in the brain and skeletal muscles, respectively.

**Conclusion:**

Endurance training induces organ-specific regulation of inflammatory and hypoxia-related proteins. The differential responses of TNF-α isoforms suggest distinct roles of local inflammatory signaling in exercise-induced adaptations. Of particular interest is the observed anti-inflammatory effect of endurance training in the brain, indicating distinct regulatory mechanisms within the CNS.

## Graphical Abstract

**Figure:**
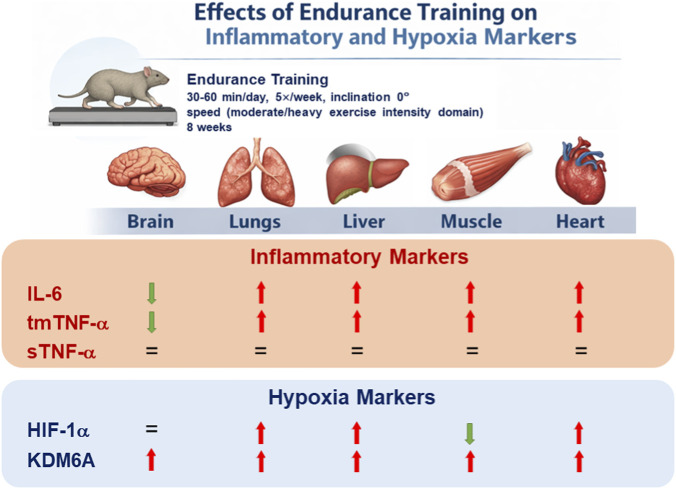

## 1 Introduction

It is well documented that physical exercise is potent at increasing the serum levels of several cytokines in humans (Legård et al., 2019; Pedersen, 2019). Over recent decades, two pro-inflammatory cytokines, interleukin 6 (IL-6) and tumor necrosis factor–alpha (TNF-α) (Tilg et al., 1997; Scheller et al., 2011), have garnered increasing attention for their roles during physical exercise (Legård et al., 2019; Pedersen, 2019; Petersen and Pedersen, 2005; Chow et al., 2022; Kistner et al., 2022). It has been shown that sustained exercise at moderate-to-heavy intensity most frequently leads to increased IL-6 in the blood (serum/plasma), which, in the exercise context, is anti-inflammatory and has several metabolic effects (Legård et al., 2019; Pedersen, 2019; Petersen and Pedersen, 2005; Kistner et al., 2022; Jønck et al., 2025). In contrast, strenuous exercise can increase both IL-6 and TNF-α in the blood in humans (Espersen et al., 1990; Dufaux and Order, 1989; Ostrowski et al., 1999; Gleeson et al., 2011), especially when performed under hypoxic conditions (Khalafi et al., 2023). Notably, several studies employing various experimental models have provided evidence confirming the link between hypoxia and inflammation (Low et al., 2025).

It has been show that exercising muscles release IL-6 (Ostrowski et al., 1998), but surprisingly still very little is known on the effect of physical training on the IL-6 and TNF-α protein expression in some vital body organs animals/humans. Therefore, in the present study, we aimed to examine the impact of 8 weeks endurance training on the IL-6 and TNF-α protein expression in the lungs, brain, liver, and hind limbs muscles of rats. To extend current knowledge on the role of hypoxia in inflammation during exercise, the present study also aimed to examine the relationship between potential training-induced increases in IL-6 and TNF-α protein expression in various organs and the training-induced levels of two tissue hypoxia markers, namely, hypoxia-inducible factor-1α (HIF-1α) (Semenza, 2000; Lee et al., 2019) and the lysine (K)-specific demethylase (KDM6A) (Chakraborty et al., 2019).

To our knowledge, this study is the first to evaluate the effects of endurance training on the two isoforms of the pro-inflammatory cytokine TNF-α—transmembrane (tmTNF-α) and soluble (sTNF-α) (Ubah et al., 2025)—in several vital organs in rats (lungs, brain, hindlimb skeletal muscle, and heart). TNF-α is synthesized as tmTNF-α (26 kDa) (Mohler et al., 1994; Moss et al., 1997; Black et al., 1997), after which proteolytic processing by ADAM metallopeptidase domain 17 (ADAM17/TACE) releases sTNF-α (17 kDa) (Ubah et al., 2025). Although both tmTNF-α and sTNF-α are biologically active, recent studies indicate that they exert distinct functions (Ubah et al., 2025).

In this study, we hypothesized that: (i) endurance training might upregulate the IL-6 protein levels not only in skeletal muscles, as previously shown (Ostrowski et al., 1998), but also in vital organs (the lungs, brain, liver, hindlimb skeletal muscle, and heart); (ii) endurance training might also increase TNF-α protein levels in these organs; and (iii) increases in pro-inflammatory cytokine protein levels (IL-6 and TNF-α) in various organs would be accompanied by increased protein levels of the hypoxia-related proteins HIF-1α and KDM6A in these organs.

## 2 Materials and methods

### 2.1 Endurance training

This study used 16 four-month-old male Wistar rats, which were randomly assigned to the endurance-trained group (*n* = 8) or the non-trained control group (*n* = 8). All experimental procedures, including animal handling and housing conditions, were conducted in accordance with Directive 2010/63/EU of the European Parliament and of the Council of 22 September 2010 on the protection of animals used for scientific purposes. The study protocol was reviewed and approved by the Local Ethical Committee for Animal Experiments in Poznan, Poland (approval no. 15/2013). Throughout the experiment, all reasonable measures were taken to minimize animal suffering.

#### 2.1.1 Training program

The endurance training program was conducted over 8 weeks and followed a previously described protocol (Zoladz et al., 2016). The training was conducted five times per week using a standard treadmill for small rodents (Exer 3/6 M Treadmill, Columbus Instruments, Columbus, OH, USA). The running belt was placed horizontally an inclination of 0°. In the first week of the training, rats were familiarized with running on the treadmill at various velocities (20–30 m min^−1^) during 20–30 min running sessions. At the end of the first week, the duration of the training session was 40 min. In the first 2 weeks of training, the basal running velocity was 30 m min^−1^, but every 10 min it was increased to 40 m min^−1^ for approximately 20 s. After completing the fourth week of training, the duration of training sessions was increased to 60 min. The basal running velocity at this stage of training was set to 30 m min^−1^, and approximately every 10 min it was increased up to 40 m min^−1^.The duration of the higher speed was gradually increased: from 20 s in the sixth week to approximately 40 s in the last week of training. At the end of the training period, 22–24 h after the last training session, rats were euthanized by decapitation, with every effort made to minimize suffering. No anesthetics were used to avoid their effects on mitochondrial function.

### 2.2 Tissue collection and homogenization

Experimental steps were performed at 4 °C. Hearts, livers, lungs, cerebral cortex samples, and hindlimb skeletal muscles were rapidly excised and repeatedly rinsed in isolation buffer A containing 50 mM Tris hydrochloride (Tris-HCl, pH 7.2), 100 mM sucrose, and 0.5 mM ethylenediaminetetraacetic acid (EDTA). After removal of visible connective tissue and large blood vessels, the tissues were finely minced, and residual blood was removed by repeated decantation.

Tissues were homogenized in isolation buffer B composed of 50 mM Tris-HCl (pH 7.2), 100 mM sucrose, 100 mM potassium chloride, 1 mM monopotassium phosphate, 0.5 mM EDTA, and 0.1 mM ethylene glycol-bis(β-aminoethyl ether)-N,N,N′,N′-tetraacetic acid. Skeletal muscle samples were homogenized using a Polytron homogenizer (T18 basic; IKA-Werke GmbH & Co. KG, Staufen, Germany) with three 2-s pulses at 80% power, whereas heart, liver, lung, and brain tissues were homogenized using glass or Teflon pestles. The tissue extracts were centrifuged at 900 *g* for 10 min, and the total protein concentration was determined by the Bradford assay using bovine serum albumin as a reference standard.

### 2.3 Immunodetection of protein levels

#### 2.3.1 Western blot analysis

Protein concentrations in tissue homogenates were adjusted to ensure equal loading based on actin as a loading control, as actin levels did not differ between control and exercised groups. Equalized protein amounts (20 or 40 µg per lane) were loaded onto gels and separated by sodium dodecyl sulfate–polyacrylamide gel electrophoresis (SDS–PAGE) at 140 V using 11.5% acrylamide gels for TNF-α and IL-6 detection or 7.5% acrylamide gels for HIF-1α and KDM6A detection. Molecular mass was estimated with the PageRuler Prestained™ Protein Ladder (Thermo Fisher Scientific, Waltham, MA, USA). Proteins were transferred using the Trans-Blot Turbo system at 7.5 mA/cm^2^ membrane area for 7 min (TNF-α and IL-6) or 10 min (HIF-1α and KDM6A) in transfer buffer containing 16.1 mM glycine, 48 mM Tris, 0.0375% SDS, and 20% ethanol, pH 9.2. Membranes were blocked for 35 min in 4% skimmed milk in TBST (15 mM Tris-HCl, 120 mM NaCl, 0.005% Tween-20, pH 7.6), followed by 60 min incubation with primary antibodies diluted in 3% protease-free bovine serum albumin (BSA) in TBST.

Primary antibodies against rat TNF-α (ab205587; 1:1000; 17 and 26 kDa) and IL-6 (ab9324; 1:2000; 26 kDa) were obtained from Abcam (Cambridge, UK). Primary antibodies against HIF-1α (PA5-85494; 1:1000; 110–140 kDa) and KDM6A (PA5-68598; 1:1000; 140 kDa) were obtained from Thermo Fisher Scientific (Waltham, MA, USA). Primary antibodies against actin (CP01; 1:1000; 42 kDa) were obtained from Merck (Darmstadt, Germany).

After incubation with the primary antibody, the membranes were washed three times for 3 min in TBST and then incubated for 30 min with secondary antibodies diluted in prewarmed (37 °C) 0.2% skimmed milk in TBST. The membranes were then washed three times for 10 min in TBST and three times for 1 min in TBS.

#### 2.3.2 Visualization

Immunoreactive bands were visualized using SuperSignal West Pico PLUS Chemiluminescent Substrate (Thermo Scientific) and imaged using a G:Box Chemi XR5 system (Syngene). Signal intensities were quantified using ImageJ software (U.S. National Institutes of Health, Bethesda, MD, USA) and normalized to actin levels. Uncropped images with corresponding protein loading controls are shown in Supplementary Figures S1, S2.

### 2.4 Statistical analysis

Statistical analyses were performed using Statistica (version 14.0; TIBCO Software, Santa Clara, CA, USA), and a *p* < 0.05 was considered statistically significant. Data are presented as the mean ± standard deviation (SD) across tissue samples obtained from 6–8 animals per experimental group. An *a priori* power analysis was performed based on previous experimental data. The planned sample size (*n* = 8 per group) was justified by the observed effect size and the high homogeneity of the experimental groups. With a mean intra-group standard deviation (SD) of approximately 18% and observed changes in protein expression reaching 26% or more, the estimated Cohen’s d was approximately 1.43, indicating a large biological effect. For this effect size, the projected statistical power for *n* = 8 per group was approximately 0.76–0.80 (α = 0.05, two-tailed), i.e., close to the conventional 0.80 threshold. In accordance with the 3Rs principle, this sample size was selected to balance statistical sensitivity with the ethical imperative to minimize animal use. The final analyzed groups were reduced to *n* = 6 due to technical exclusions (e.g., blood contamination of tissue homogenates).

To increase the reliability of the analyses and minimize technical variability inherent to immunoblotting, samples from sedentary and exercise-trained animals were pair-matched based on comparable levels of blood contamination and similar initial total protein concentrations. Each matched control–trained pair was always loaded onto adjacent lanes of the same polyacrylamide gel to reduce inter-lane and inter-gel variability. Densitometric data were analyzed as paired ratios of protein band intensities in trained relative to control animals, with signals normalized to actin as a loading control. Trained/control ratios were log2-transformed prior to statistical analysis. The effect of training on protein expression was assessed using a two-tailed one-sample *t*-test (α = 0.05). Assumptions of approximate normality were evaluated using Q–Q plots and supplemented, where appropriate, by the Shapiro-Wilk test. Differences in training-induced responses among organs were assessed using one-way ANOVA performed on the log2-transformed responses across tissues. When a significant main effect was detected, pairwise comparisons were conducted using Tukey’s honestly significant difference (HSD) *post hoc* test to correct for multiple comparisons.

## 3 Results

### 3.1 Endurance training induces a pro-inflammatory cytokine response in peripheral tissues without TNF-α activation and stimulates an anti-inflammatory response in the brain

**FIGURE 1:**
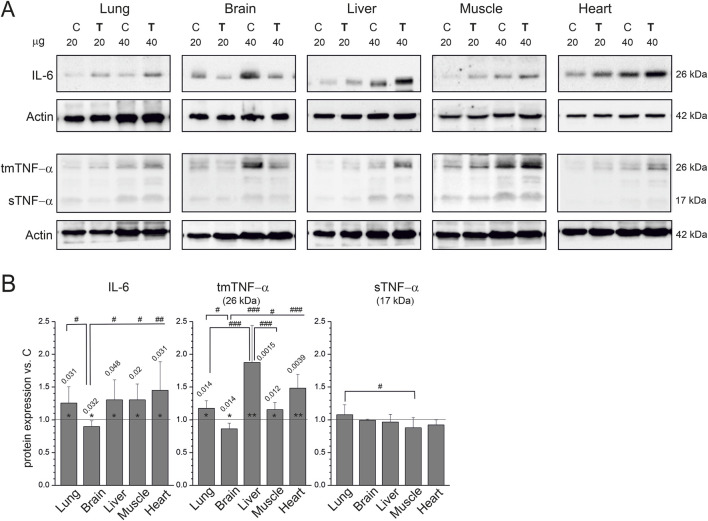
Inflammatory markers in tissues from non-trained (C) and endurance-trained (T) rats. **(A)** Representative western blots (cropped blots, 20 or 40 µg of protein per lane). **(B)** Analysis of protein expression levels normalized to actin, presented as the mean ± SD across six samples (*n* = 6). Bars representing statistically significant changes vs. control within individual organs are annotated with exact *p* values; significance: *, *p* < 0.05; **, *p* < 0.01. Statistically significant differences in protein expression responses between organs, identified by Tukey’s HSD *post hoc* test, are indicated by #: #, *p* < 0.05; ##, *p* < 0.01; ###, *p* < 0.001. Abbreviations: IL-6, interleukin 6; sTNF-α, cleaved, soluble tumor necrosis factor–alpha (17 kDa); tmTNF-α, transmembrane tumor necrosis factor–alpha (26 kDa).

To date, changes in the levels of inflammatory markers in response to endurance training have been investigated primarily in plasma at the protein level and in tissues at the transcript level. We detected protein levels of two inflammatory markers, IL-6 and TNF-α, in the hearts, livers, brains, lungs, and skeletal muscles of sedentary and trained rats (Figure 1). Compared with control animals, IL-6 levels decreased slightly in the brain (12%) and increased in the hearts, livers, lungs, and skeletal muscles (25%–45%) of trained rats (Figure 1).

We detected TNF-α immunoreactive bands with molecular masses corresponding to the soluble (cleaved) form (sTNF-α, 17 kDa) and the membrane-bound form (tmTNF-α, 26 kDa) (Figure 1). In untrained and trained rats, the amount of tmTNF-α was higher than the that of cleaved soluble form (sTNF-α) in all tissues examined. In the heart, both forms were present in low amounts. In trained rats compared to untrained rats, changes in tmTNF-α levels were associated with changes in IL-6 levels, increasing in the heart, liver, lung, and skeletal muscle, and decreasing in the brain (15%). In contrast, sTNF-α remained unchanged in all tissues, indicating that training likely altered TNF-α processing without up-regulating the potent pro-inflammatory sTNF-α isoform.

One of the most compelling findings is the decrease in IL-6 and tmTNF-α levels in the brain (12% and 15%, respectively). Although the percentage decreases in both markers measured in the brain appeared modest, standardized effect size analysis revealed a robust biological impact. Hedges’ *g* showed large effect sizes for both IL-6 (*g* = −1.209, 95% *CI* [−2.356, −0.051]) and TNF-α (*g* = −1.678, 95% *CI* [−2.923, −0.432]), supporting a substantial biological effect.

### 3.2 Endurance training increases HIF-1α levels in the lungs, liver, and heart, decreases them in skeletal muscle, and does not affect them in the brain

**FIGURE 2:**
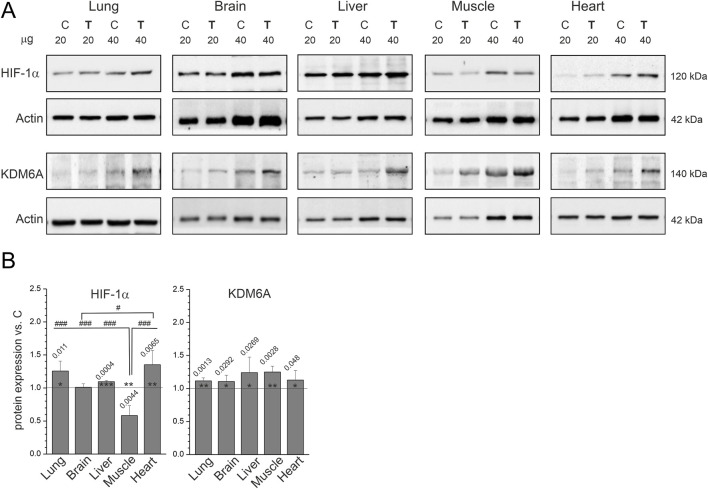
Markers of hypoxia in tissues from non-trained (C) and endurance-trained (T) rats. **(A)** Representative western blots (cropped blots, 20 or 40 µg of protein per lane). **(B)** Analysis of protein expression levels normalized to actin, presented as the mean ± SD across six samples (*n* = 6). Bars representing statistically significant changes vs. control within individual organs are annotated with exact *p* values; significance: *, *p* < 0.05; **, *p* < 0.01; ***, *p* < 0.001. Statistically significant differences in protein expression responses between organs, identified by Tukey’s HSD *post hoc* test, are indicated by #: #, *p* < 0.05; ###, *p* < 0.001. Abbreviations: HIF-1α, hypoxia-inducible factor 1 subunit alpha; KDM6A, lysine demethylase 6A.

The protein levels of HIF-1α, a hypoxia marker (Semenza, 2000; Lee et al., 2019), were increased in the lungs, liver, and heart, decreased in hindlimb skeletal muscle, and unchanged in the brain of endurance-trained rats compared to non-trained rats (Figure 2). Although endurance training differentially affected protein levels of HIF-1α across tissues, protein levels of KDM6A, a direct cellular oxygen sensor involved in gene regulation (Chakraborty et al., 2019), were increased in all tissues of endurance-trained rats compared to non-trained rats (Figure 2). The simultaneous increase in HIF-1α and KDM6A protein levels in lung, liver, and heart at first glance suggests that endurance training may cause local/transient hypoxia in these tissues, but their increase may be part of another mechanism that has important implications for cell/organ function.

## 4 Discussion

### 4.1 Impact of endurance training on tissue hypoxia markers

The transcription factor HIF-1α (Wang and Semenza, 1995; Semenza et al., 1996; Jiang et al., 1996) plays a vital role in the cellular response to systemic changes in oxygen levels in mammals, serving as an essential mediator of oxygen homeostasis (Semenza, 2000; Lee et al., 2019). HIF-1α activates genes involved in angiogenesis, energy metabolism, erythropoiesis, cell survival, vascular remodeling, and vasomotor regulation (Semenza, 2000; Lee et al., 2019). Recently, HIF-1α and its downstream effectors have emerged as novel therapeutic targets for treating injuries to various organs, including the heart, lung, liver, and kidney (Lee et al., 2019). Although usually associated with hypoxia, HIF-1α can be detected under normoxic conditions in many organs, especially in pathological conditions (Lee et al., 2019). Physical activity has been shown to increase HIF-1α expression in skeletal muscle under both normoxic and hypoxic conditions (Ameln et al., 2005; Van Thienen et al., 2016), although its role in training-induced muscle adaptation remains debated (Lundby et al., 2009).

Surprisingly little is known about the impact of endurance training on HIF-1α expression in various organs. In this study, 8 weeks of endurance training reduced HIF-1α levels in skeletal muscle. This finding supports the notion that long-term endurance training attenuates the acute HIF-1α response, likely through negative regulatory mechanisms that promote oxidative stress as part of a local physiological adaptation to exercise (Lindholm and Rundqvist, 2016). Interestingly, mice lacking HIF-1α in skeletal muscle failed to induce glycolytic genes and instead shifted toward aerobic metabolism, which improves endurance capacity (Mason et al., 2004). Consistent with these findings, Mason and Johnson concluded that removal of HIF-1α resulted in an adaptive response in skeletal muscle resembling endurance training, providing evidence that HIF-1α suppresses mitochondrial biogenesis in normal tissue (Mason and Johnson, 2007). Interestingly, a clear tissue-specific pattern emerged when the training-induced changes in HIF-1α levels observed in this study were compared with the changes in maximal cytochrome *c* oxidase (COX) activity in the same rats reported previously (Galganski et al., 2025). In skeletal muscle, a marked increase in COX activity (∼70%) (Galganski et al., 2025) was accompanied by a decrease in HIF-1α levels (Figure 2), whereas in the heart, increased HIF-1α levels were not accompanied by increased COX activity (Galganski et al., 2025). Thus, our findings support a potential link between HIF-1α expression and mitochondrial biogenesis (Mason et al., 2004) in skeletal muscle and heart during endurance training, as reflected by COX activity (a marker of oxidative phosphorylation activity (Zoladz et al., 2014a)).

Surprisingly, in this study found that endurance training increased HIF-1α levels in the lungs, liver, and heart but had no effect on them in the brain (Figure 2). Although the classical explanation would attribute these increases to local training-induced hypoxia (Semenza, 2000; Lee et al., 2019), an alternative explanation suggests that HIFs may not merely respond to hypoxic stress but also prevent its onset by regulating electron flux between catabolic substrates and oxygen (Arias et al., 2025). Namely, it has been reported that in the absence of HIFs, the NAD+/NADH cycle becomes easily saturated with electrons when oxygen levels are low or metabolic supply is high (Arias et al., 2025). Therefore, the increased HIF-1α levels observed in the lungs, liver, and heart (Figure 2) may result from transient ischaemia/hypoxia of the internal organs, occurring during changes in blood flow distribution during physical exercise and/or may be interpreted an organ response aimed at maintaining energy homeostasis, as recently proposed by Arias et al. (Arias et al., 2025). Therefore, further studies are needed to clarify the role of HIF-1α in the training responses of different organs and to elucidate their physiological significance. Interestingly, it was recently shown that HIF-1α signaling also controls collagen synthesis and modification in chondrocytes at the metabolic level (Stegen et al., 2019), suggesting a broader role in training-induced adaptation of connective tissues.

KDM6A, a histone demethylase associated with cell differentiation, tumor progression, and hypoxia signaling (Chakraborty et al., 2019; Tran et al., 2020; Singh et al., 2026), is less well studied in the context of endurance training. In this study, KDM6A levels increased in all examined tissues except the heart (Figure 2). However, training-induced changes in KDM6A levels did not always correspond to changes in HIF-1α levels. Although both increased in the lung and liver, HIF-1α decreased in skeletal muscle, while KDM6A instead increased. Currently, the significance of the upregulation of KDM6A in various organs during exercise remains unclear. However, emerging evidence links KDM6A-dependent epigenetic regulation to neuronal regeneration and brain function during aging (Yang et al., 2025; Davis et al., 2020), warranting further investigation of its role in exercise-induced adaptation in the central nervous system (CNS).

### 4.2 Impact of endurance training on tissue inflammation markers

In this study, endurance training increased IL-6 levels in all examined organs except the brain, where they were decreased (Figure 1). IL-6 is a pleiotropic cytokine that primarily exerts pro-inflammatory effects in most tissues (Tilg et al., 1997; Scheller et al., 2011). However, when released from contracting skeletal muscles, it acts as a myokine with anti-inflammatory and metabolic-regulating effects (Legård et al., 2019; Pedersen, 2019; Petersen and Pedersen, 2005; Chow et al., 2022; Kistner et al., 2022; Ostrowski et al., 1998). Although the functional implications of increased IL-6 levels in the lung, liver, and heart (Figure 1) remain unclear, it is likely that, as in skeletal muscle, IL-6 contributes to improved organ function during exercise training.

A novel and important finding of this study is the differential regulation of TNF-α isoforms by endurance training. While sTNF-α levels remained unchanged in all examined tissues, tmTNF-α levels increased in the lung, liver, skeletal muscle, and heart but decreased in the brain (Figure 1). To our knowledge, this is the first demonstration that TNF-α isoforms respond differentially to endurance training in a tissue-specific manner. Both sTNF-α and tmTNF-α have biological functions, but their scope of action differs because sTNF-α acts at sites distant from TNF-α–producing cells, whereas tmTNF-α acts through cell-to-cell contact, playing a key role in local inflammation (Ubah et al., 2025). The tmTNF-α isoform may exert various effects in organs, including inducing apoptosis, participating in cardiac remodeling (Igaki et al., 2002; Dibbs et al., 2003; You et al., 2021), and mediating the promotion of cell proliferation and survival (Qu et al., 2017). Moreover, it has recently been reported that, unlike the pathogenic effects of sTNF-α in sepsis, tmTNF-α acts as an anti-inflammatory agent (Li et al., 2019).

The observed increase in tmTNF-α levels in skeletal muscle, heart, liver, and lung, accompanied by its reduction in brain and unchanged sTNF-α levels (Figure 1), indicates a dissociation between TNF-α synthesis and its proteolytic shedding. While ADAM17/TACE is the major TNF-α sheddase (Black et al., 1997), TNF-α processing involves a broader and partially redundant proteolytic network (Sisto and Lisi, 2024). TNF-α expression is primarily regulated at the transcriptional level via redox-sensitive pathways such as NF-κB and AP-1, which are activated during repeated metabolic stress (Ji, 2015). The lack of increased sTNF-α levels likely reflects insufficient activation of the shedding machinery despite increased substrate availability. The applied eight-week endurance training represents a chronic adaptive stimulus characterized by reduced basal inflammation and improved redox homeostasis (Petersen and Pedersen, 2005). Such conditions are not typically associated with robust activation of ADAM-dependent shedding, which is more strongly linked to acute inflammatory signaling (Sisto and Lisi, 2024). Additionally, mechanisms such as altered membrane microdomain organization or increased expression of endogenous inhibitors (e.g., TIMP3) may further limit TNF-α shedding despite enhanced synthesis (Amour et al., 1998).

Thus, the precise significance of the training-induced increase in tmTNF-α levels in various organs remains unknown. We hypothesize that the endurance training–induced upregulation of tmTNF-α in most of the organs examined (Figure 1) may exert regulatory effects that improve their function. The lack of influence of endurance training on the level of pro-inflammatory sTNF-α observed in this study (Figure 1) is consistent with the notion that in humans, physical activity generally does not lead to systemic inflammation but has an anti-inflammatory effect, as indicated by the decreased levels of circulating pro-inflammatory markers and cytokines (Petersen and Pedersen, 2005; Luo et al., 2024). Consistently, moderate-intensity training reduced the basal serum levels of soluble vascular cell adhesion molecule 1 (VCAM1) and TNF-α in patients with Parkinson’s disease (Zoladz et al., 2014b).

However, strenuous exercise increases levels of pro-inflammatory cytokines (Espersen et al., 1990; Dufaux and Order, 1989; Ostrowski et al., 1999; Gleeson et al., 2011). For example, strenuous exercise induced increases in TNF-α and interleukin 1 beta (IL-1β), along with a dramatic increase in the inflammation-responsive cytokine IL-6 (Ostrowski et al., 1999). Importantly, this pro-inflammatory response is balanced by the release of cytokine inhibitors (interleukin 6 receptor [IL6R/IL-1Ra], soluble TNF receptor superfamily member 1A [TNFRSF1A/TNF-R1], and TNF receptor superfamily member 1B [TNFRSF1B/TNF-R2]) and the anti-inflammatory cytokine interleukin 10 (IL-10) (Ostrowski et al., 1999). Similarly, a systematic review concluded that circulating TNF-α and IL-10 increase primarily after intense exercise, whereas pro-inflammatory cytokines IL-6 and IL-1β increase more after intense compared with moderate exercise (Cerqueira et al., 2019). A recent review concluded that exercise performed under normoxic or hypoxic conditions increases circulating IL-6 and IL-10 levels in humans, whereas only hypoxic exercise increases circulating TNF-α levels (Khalafi et al., 2023), which is consistent with the proposed role of hypoxia in inflammation development (Low et al., 2025). In this study, increased levels of HIF-1α and KDM6A (Figure 2) in selected organs were accompanied by increased tmTNF-α levels, but not by changes in sTNF-α levels (Figure 1). Thus, training-induced hypoxia-related signaling may preferentially activate tmTNF-α without affecting sTNF-α.

The role of training-induced upregulation of IL-6 and tmTNF-α in the systemic adaptation to endurance training (Figure 1) remains unclear. In patients with rheumatoid arthritis treated with IL-6 (IL-6i) or TNF-α (TNF-αi) inhibitors, physical activity increased left ventricular mass only in patients receiving TNF-αi and peak oxygen consumption (V̇O_2peak_) only in patients receiving IL-6i, suggesting differential roles of IL-6 and TNF-α signaling in determining V̇O_2peak_ in humans (Jønck et al., 2025). These findings strongly suggest that the training-induced increase in IL-6 and tmTNF-α levels observed in this study, occurring in most examined organs (the lung, liver, hindlimb skeletal muscle, and heart; Figure 1), may play a role in systemic adaptation to exercise training.

## 5 Conclusion

This study demonstrated that moderate/high-intensity endurance training induces pronounced, organ-specific alterations in inflammatory and hypoxia-related signaling pathways at the protein level. Increased tmTNF-α and IL-6 protein levels in the lungs, liver, hindlimb skeletal muscle, and heart suggest a role for local, cell-to-cell inflammatory signaling in exercise-induced organ adaptation. The observed increases may reflect adaptive physiological responses; however, this interpretation should be considered with caution, as direct markers of organ damage were not measured, limiting the ability to exclude subclinical pathological inflammation. Of particular interest is the observed anti-inflammatory effect of endurance training in the brain, indicating distinct regulatory mechanisms within the CNS. Further research is needed to clarify the significance of these responses in the context of health and exercise tolerance in animals and humans, taking into account the impact of exercise training on organ function and health.

## Data Availability

The original contributions presented in the study are included in the article/Supplementary Material, further inquiries can be directed to the corresponding authors.
