# Supplementary material for: Endurance training increases tmTNF-α and IL-6 levels in several vital organs in rats: relationship with hypoxia markers
Source: Front Mol Biosci. 2026 May 14;13:1815657. doi: 10.3389/fmolb.2026.1815657 (PMC13215900; doi:10.3389/fmolb.2026.1815657)
Supplement: Supplementary file 1 [file DataSheet1.pdf]

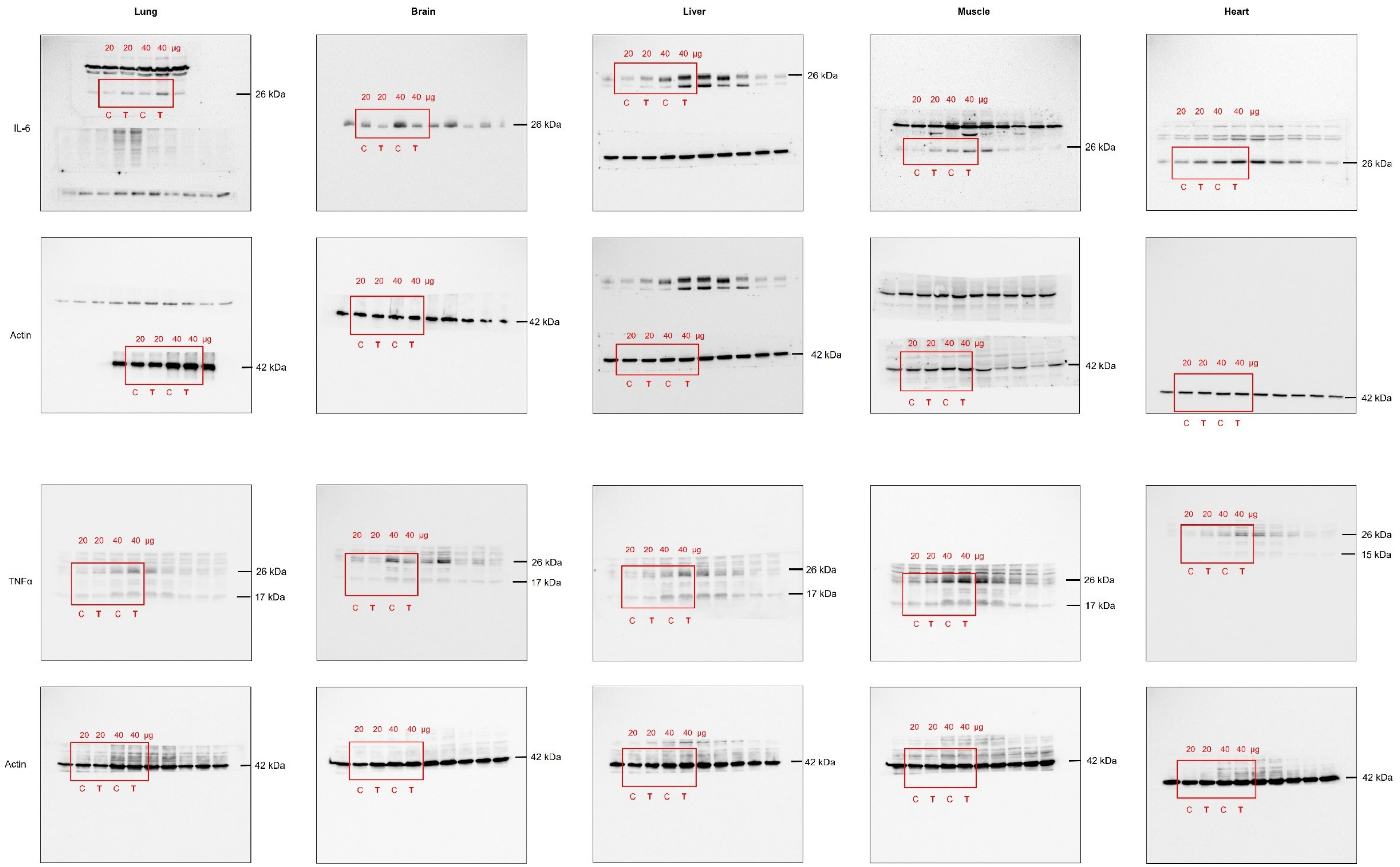

Supplementary Figure 1. Original images used for the preparation of Fig. 1. Abbreviations: IL-6, interleukin 6; sTNF-α, cleaved, soluble tumor necrosis factor–alpha (17 kDa); tmTNF-α, transmembrane tumor necrosis factor–alpha (26 kDa).

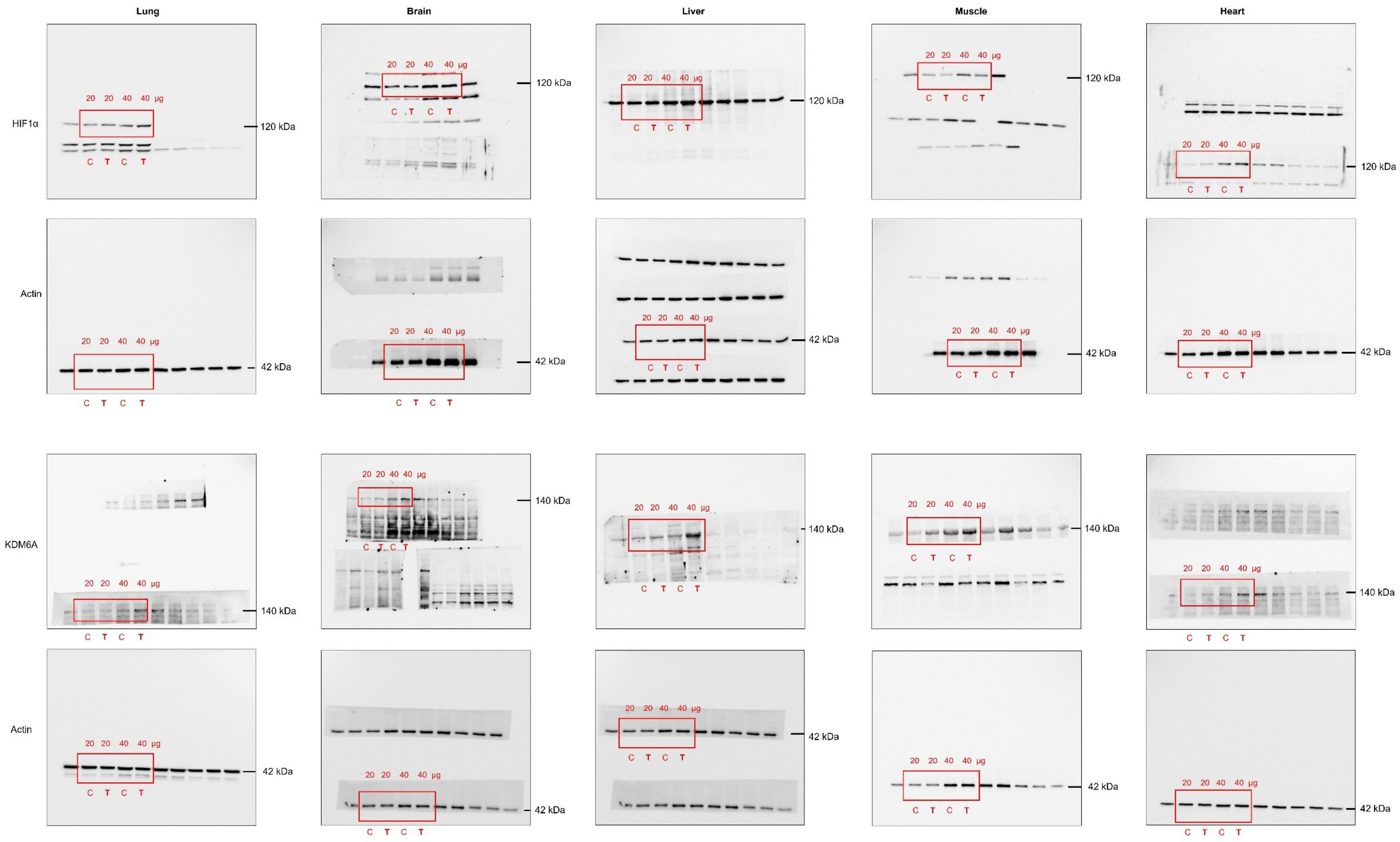

Supplementary Figure 2. Original images used for the preparation of Fig. 2. Abbreviations: HIF-1α, hypoxia-inducible factor 1 subunit alpha; KDM6A, lysine demethylase 6A.
